# Self-DNA at the Epicenter of SLE: Immunogenic Forms, Regulation, and Effects

**DOI:** 10.3389/fimmu.2019.01601

**Published:** 2019-07-10

**Authors:** Chetna Soni, Boris Reizis

**Affiliations:** ^1^Department of Pathology, New York University School of Medicine, New York, NY, United States; ^2^Department of Medicine, New York University School of Medicine, New York, NY, United States

**Keywords:** DNases, systemic lupus erythematosus, toll-like receptors, interferons, autoantibodies

## Abstract

Self-reactive B cells generated through V(D)J recombination in the bone marrow or through accrual of random mutations in secondary lymphoid tissues are mostly purged or edited to prevent autoimmunity. Yet, 10–20% of all mature naïve B cells in healthy individuals have self-reactive B cell receptors (BCRs). In patients with serologically active systemic lupus erythematosus (SLE) the percentage increases up to 50%, with significant self-DNA reactivity that correlates with disease severity. Endogenous or self-DNA has emerged as a potent antigen in several autoimmune disorders, particularly in SLE. However, the mechanism(s) regulating or preventing anti-DNA antibody production remain elusive. It is likely that in healthy subjects, DNA-reactive B cells avoid activation due to the unavailability of endogenous DNA, which is efficiently degraded through efferocytosis and various DNA-processing proteins. Genetic defects, physiological, and/or pathological conditions can override these protective checkpoints, leading to autoimmunity. Plausibly, increased availability of immunogenic self-DNA may be the key initiating event in the loss of tolerance of otherwise quiescent DNA-reactive B cells. Indeed, mutations impairing apoptotic cell clearance pathways and nucleic acid metabolism-associated genes like DNases, RNases, and their sensors are known to cause autoimmune disorders including SLE. Here we review the literature supporting the idea that increased availability of DNA as an immunogen or adjuvant, or both, may cause the production of pathogenic anti-DNA antibodies and subsequent manifestations of clinical disease such as SLE. We discuss the main cellular players involved in anti-DNA responses; the physical forms and sources of immunogenic DNA in autoimmunity; the DNA-protein complexes that render DNA immunogenic; the regulation of DNA availability by intracellular and extracellular DNases and the autoimmune pathologies associated with their dysfunction; the cytosolic and endosomal sensors of immunogenic DNA; and the cytokines such as interferons that drive auto-inflammatory and autoimmune pathways leading to clinical disease. We propose that prevention of DNA availability by aiding extracellular DNase activity could be a viable therapeutic modality in controlling SLE.

## Introduction

### Anti-DNA Antibodies as a Biomarker for SLE

Anti-DNA antibodies (Abs) are not exclusive to systemic lupus erythematosus (SLE or lupus), yet, their persistence in serum is the most reliable serological marker for lupus diagnosis (1–4). High titers of anti-DNA Abs directly correlate with disease activity (3, 5), predictions of lupus flares (6, 7), hypocomplementemia (8), and proliferative lupus nephritis (9, 10). 70–80% of SLE patients have detectable levels of anti-DNA Abs, of which ~45–50% have high titers (3, 8, 11). This is in contrast with anti-DNA Ab- positive non-SLE patients with rheumatoid arthritis (RA), scleroderma, vasculitis, tuberculosis, autoimmune hepatitis, viral hepatitis or cancer, where the titers are predominantly low-to-moderate (3, 8). Additionally, a fraction of aged healthy individuals also have anti-DNA Abs but rarely at high titers (12). The correlation of high titers of anti-DNA Abs with SLE disease severity is indicative of a requirement for the persistent availability of DNA as an immunogen. Additionally, many pathological conditions including infections, and cancer can induce anti-DNA Abs which invokes a status for DNA as a readily available adjuvant associated with various proteins under different conditions.

### B Cells in Anti-DNA Responses

Rheumatic diseases like SLE, RA, Sjogren's syndrome, vasculitis, antiphospholipid syndrome etc., which cause development of anti-DNA Abs in several patients, are driven by B cells (13, 14). Moreover, DNA-specific B cells can readily expand in all individuals upon exposure to microbial DNA (4). In healthy individuals, the microbial DNA-specific B cell expansion is transient. However, under autoimmune conditions, the bacterial DNA-reactive B cells also recognize self-DNA and are retained after the infection is cleared (15). It is therefore of clinical relevance to understand the conditions which cause the persistence of DNA-reactive B cells in autoimmune diseases like SLE. Toward this goal, significant advances have been made in the area of B cell biology to understand the regulation of autoreactive B cells. A recent comprehensive review on B cell genetic risk factors involved in SLE highlighted the importance of examining specific B cell subsets for better targeted therapeutic intervention (16). The major B cells subsets implicated in anti-DNA antibody production include germinal center (GC) B cells that produce long-lived plasma/ memory cells and the extrafollicularly generated short-lived plasmablasts (17, 18). Several studies in mice outline a significant role of the extrafollicular pathway in anti-DNA/ chromatin Ab production, showing that B cells can undergo both isotype switching and affinity maturation outside of the GCs (19–22). Notably, expansion of extrafollicular B cells in active human SLE patients has also been reported (23, 24). In a recent study, specific subsets of B cells involved in the extrafollicular pathway of autoantibody production in SLE were defined in patients with active disease (25). Unlike the GC pathway, the absence of extrafollicular tolerance checkpoints might explain the preferential emergence and amplification of anti-DNA responses via the extrafollicular route.

In accordance with the predominantly short-lived nature of DNA-reactive B cells, B cell targeting therapies like Rituximab (anti-CD20) and Belimumab (anti-BAFF) have been partially effective in SLE treatment (13, 14, 26). There was a modest yet significant reduction of SLE disease severity in patients with serologically and clinically active SLE upon treatment with Belimumab (Benlysta), alongside standard therapy (7, 27–29). Notable observations from phase III Belimumab trials BLISS−52 and BLISS−76 (30, 31) were that increased anti-DNA Ab titers predicted lupus flares (6, 7), while successful treatment resulted in reduced anti-DNA Abs (29), positively correlating anti-DNA Abs with disease manifestations. Although anti-B cell therapies are promising (14, 32), there remains great variability in the reduction of autoantibodies and disease severity upon treatment, in part due to the variable B cell subsets involved in antibody production. Additionally, most patients receive supplemental concurrent administration of corticosteroids that have several adverse side effects, including infections, hypertension, hyperglycemia, osteoporosis, cataracts, glaucoma, and cognitive impairment (33, 34). Therefore, effective treatment of SLE with minimal side effects requires newer approaches and interventions in addition to and beyond B cell-targeted therapy.

### T Cells in Anti-DNA Responses

**Figure 1:**
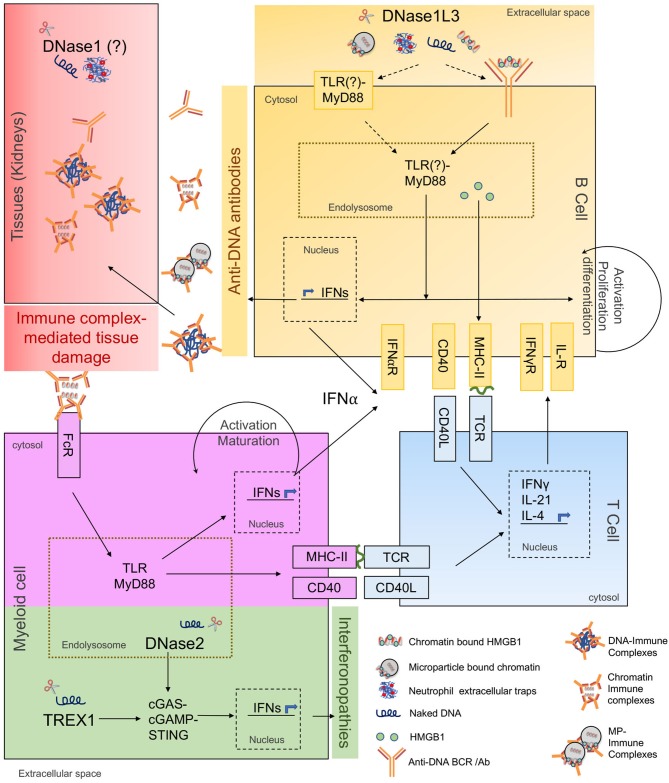
Cellular and molecular responses to extracellular and intracellular DNA. The schematic shows involvement of extracellular DNases in anti-DNA responses/ SLE pathogenesis and intracellular DNases in interferonopathies. The major molecular pathways of autoantibody and autoinflammatory responses are highlighted in different colors as described below. **Yellow**: Primary cellular and molecular pathways of anti-DNA Ab production. DNase1L3-deficiency increases availability and uptake of cfDNA (naked DNA, NET-DNA, cell-free chromatin, and microparticle-associated chromatin), along with associated proteins potentially through self-reactive BCRs or through cell-surface TLRs. Internalized self-DNA causes TLR-MyD88 dependent B cell activation, differentiation, IFN production, and presentation of cfDNA-associated peptides to T cells. **Blue**: T cells help in anti-DNA Ab production. Costimulatory and cognate MHC-TCR interactions between DNA-reactive B and T cells stimulate activation, proliferation, and differentiation of B cells into anti-DNA Ab secreting cells. **Purple**: Amplification of anti-DNA Abs through myeloid cell help. Anti-DNA antibodies accumulate and form immune complexes with cfDNA which are internalized through Fc-receptors on myeloid cells i.e., DCs, pDCs, macrophages, further inducing IFN production through TLR-MyD88 pathway. Myeloid cells also present self-antigen to T cells further amplifying the B-T cell interaction loop and anti-DNA Ab production. **Red**: Undigested DNA promotes IC formation and deposition in target organs. DNase1 expressed in kidneys digests locally produced apoptotic cell-derived DNA. IC-formation is enhanced in the presence of extracellular DNA. ICs deposit in kidneys causing immune complex-mediated tissue damage. **Green**: DNases and signaling pathways regulating interferonopathies. DNase2 cleaves endocytosed apoptotic cell-derived DNA while TREX1 cleaves cytosolic DNA. Absence of DNase2 and TREX1 trigger activation of cGAS-STING pathway causing IFN production leading to interferonopathies. DNase2 and TREX1 do not directly contribute to anti-DNA antibody production.

Along with B cells, the generation, and amplification of anti-DNA antibodies requires a T-cell dependent antigenic stimulation process, which indicates that anti-DNA antibody production is not just a consequence of polyclonal stimulation of immune cells. Indeed, autoreactive T cell clones have been identified in mice (35) and humans (36–38) and are essential for the amplification of autoreactive B cells (Figure 1). A subset of CD4^+^ T cells expressing high CXCR5, ICOS, and PD-1, named follicular helper T cells (Tfh) are particularly implicated in several autoimmune diseases. Tfh promote the generation of germinal center-driven anti-DNA Abs in several lupus mouse models by providing key cytokines like IL-21 and IL-4 to B cells in the germinal centers (39–41). Likewise, a subset of SLE patients have increased numbers of CD4^+^CXCR5^+^ICOS^hi^PD-1^hi^ circulating T cells, resembling mouse Tfh cells (39, 42, 43). Another distinct population of helper T cells has also been identified in the generation and amplification of anti-DNA/ chromatin responses through the extrafollicular pathway in mice (44–46), and more recently in SLE patients (47). Given the pleiotropic roles of T cells as B cell helpers (Tfh), cytokine producers (Th1, Th17) and suppressors of autoimmunity (Tregs) in SLE, it is no surprise that several T-cell targeted therapies are in use and/ or under investigation for lupus (48).

### pDCs in Anti-DNA Responses

In addition to the direct role of B and T cells in anti-DNA Ab production, high serum type-I interferon levels and activity directly correlate with high anti-DNA Ab titers in SLE patients (49–51). Plasmacytoid dendritic cells (pDCs) are considered as professional IFN-I producing cells and are implicated in autoimmunity (52, 53). Stage III-IV lupus nephritis (LN) patients also show increased infiltration of pDCs in kidneys (54). Consistently, pDC depletion in BXSB and B6. *Nba2* models of SLE ameliorated disease (55, 56). Furthermore, functional impairment of pDCs by monoallelic deletion of *Tcf4* was sufficient to reduce autoantibody production and disease manifestations in two genetic mouse models (57). Clearly, the role of pDCs in autoimmunity is evident but their precise role in anti-DNA antibody production needs further investigation. In humans, pDCs were shown to promote B cell differentiation into plasmablasts/ plasma cells by producing IFNα and IL-6 *in vitro* (58), while activated human B cells were able to induce IFNα production by pDCs (59). In another study, pDCs from healthy subjects promoted the expansion of IL-10 producing regulatory B cells through IFNα, while pDCs from SLE patients did not (60). This evidence for a reciprocal interaction between B cells and pDCs with the involvement of IFNα, warrants further investigation of the role of pDCs in anti-DNA antibody production.

Taken together, the generation of anti-DNA Abs in SLE requires the activation and interaction of several key immune cell types, depicted in Figure 1. In the following sections we will review what we know so far about the forms of antigenic DNA, its regulation and sensing, and the effector responses that drive anti-DNA Ab production.

### Immunogenic DNA: Sources and Protein Partners

DNA by itself is a weak antigen compared to macromolecules like proteins, lipids, and glycans. However, certain nucleotide sequences and structural determinants can be immunogenic. Anti-DNA Abs to specific bacterial DNA are present in healthy individuals and do not react with other bacterial or endogenous DNA (61). On the other hand, antibodies to bacterial DNA in SLE patients cross react with all DNA irrespective of its source (61–63). Such promiscuity of anti-DNA Abs in SLE patients could be explained through: (1) positive selection of BCR clones recognizing common determinants of DNA, e.g., phosphodiester backbone due to B cell tolerance checkpoint defects; (2) epigenetic/ structural modification of endogenous DNA through chemical modifications or interactions with DNA-binding proteins; or (3) the excessive availability of immunogenic cell-free DNA (cfDNA) due to clearance or DNA digestion defects. Overall, it is likely that the availability of modified immunogenic DNA to DNA-reactive B cells precipitates SLE-associated pathogenic anti-DNA responses (Figure 1). cfDNA is detectable in the serum and plasma of healthy subjects (64), while its levels increase in conditions associated with excessive cell death, e.g., pulmonary embolism, mechanical, or drug induced injury/ trauma, cancer, pregnancy, sepsis, organ transplantation, RA and SLE (65, 66), summarized in Table 1. The common forms of cell death that cause cfDNA release include apoptosis, necrosis, and NETosis.

**Table 1:** Autoimmune responses to extracellular DNA—Antigens, regulators, and sensors.

**cfDNA-Association or source**	**Generated through**	**Associated proteins**	**Sensitive to**	**Sensors**	**Associated pathologies**	**Key References**
Chromatin	Apoptosis Necrosis NETosis Pyroptosis	Histones HMGB1	DNase1L3 > Dnase1	TLR9 TLR2 TLR4 RAGE	SLE RA Sjogren's Syndrome	(67–69)
Microparticles	Apoptosis Cellular-activation Necrosis	Histones HMGB1 G3BP	Dnase1L3	MyD88-signaling pathway	SLE HUVS	(67, 70–73)
Neutrophil Extracellular Traps (NETs)	NETosis	Histones HMGB1 LL-37 MPO HNP Other granular proteins	Dnase1L3 and Dnase1	TLR4 TLR9	SLE RA Psoriasis	(74–82)
Mitochondrial	NETosis	TFRAM	Dnase1L3 (?) Dnase1 (?)	TLR9 RAGE	SLE	(78, 83, 84)
Bacterial	Infection	Curli Amyloid ERV gp70 β_2_GPI	DNase1 Dnase1L3 (?)	TLR2/ TLR9 ?	SLE AIH	(85–87)
Cancer	Tumor cell apoptosis, Necrosis	?	Dnase1 Dnase1L3 (?)	?	Anti-DNA Abs ?	(3, 65, 66, 88, 89)
Fetal	Apoptosis of fetal cells	?	DNase1L3	?	?	(66, 90, 91)

*ERV gp70, Endogenous retrovirus glycoprotein 70; HMGB1, High mobility group box 1; G2RB, galectin 3 binding protein; LL-37, cathelicidin-derived antimicrobial peptide; MPO, Myeloperoxidase; TFRAM, Transcription factor A-mitochondria; β_2_GPI, β_2_ Glycoprotein I; SLE, Systemic lupus erythematosus; AIH, Autoimmune hepatitis; HNP, Human Neutrophil protein; HUVS, Hypocomplementemic urticarial vasculitis syndrome; ?, unknown*.

### Neutrophil Extracellular Traps

NETosis is a form of neutrophil cell death involving release of neutrophil extracellular traps—NETs (92). NETs are released through a process of nuclear decondensation followed by either slow (lytic) or rapid (non-lytic) release of chromatin studded with neutrophil granular proteins. The complex biology of NETs/ NETosis and its roles in antimicrobial immunity, pathological conditions like allergic asthma, vasculitis, RA, psoriasis, and SLE were recently comprehensively reviewed (74). Increased NETosis was identified in kidney and skin biopsies from SLE patients with lupus nephritis and cutaneous SLE, respectively (93). Moreover, a positive correlation was observed in SLE patients with reduced NET-associated DNA (NET-DNA) degradation and lupus nephritis (94). The pathogenic effects of NETs in psoriasis (75) and SLE (76, 77) have been attributed to their stimulatory activity on pDCs, wherein nucleic acid-mediated TLR9/7 stimulation causes type I IFN secretion, which in turn potentiates the autoinflammatory loop (76–78).

The stimulatory NET components are a composite of neutrophil genomic DNA (gDNA), mitochondrial DNA (mtDNA) and neutrophil granular proteins, which are interferonogenic (78, 83, 84). Like gDNA MtDNA is associated with DNA-binding proteins to form complexes called nucleoids, akin to chromatin. Transcription factor A-mitochondria (TFAM), is a high-mobility group (HMG) protein involved in the compaction of mitochondrial DNA into nucleoids. Unlike other cells, damaged mtDNA in neutrophils is not degraded through “mitophagy”; instead, damaged-unoxidized mtDNA is decondensed and expelled, while oxidized mtDNA (ox-mtDNA) is degraded through lysosomes within neutrophils or after uptake by macrophages. Both these pathways are non-inflammatory in healthy individuals (95). However, in several SLE patients, due to the blocking effect of anti-RNP Abs or IFNs on TFAM, neutrophil-mtDNA is unable to dissociate from nucleoids, hence ox-mtDNA is retained within the neutrophils and expelled with NETs, which induces the production of type-I IFNs through pDCs (78). Indeed, in about 50% of SLE patients (*n* = 14) with anti-RNP Abs, ox-mtDNA is present, and so are antibodies to it (78). Increased NETosis (77, 84) and increased anti-mtDNA Abs are associated with increased anti-dsDNA, IFN-signature and disease activity index in SLE patients (83), indicating an important role of neutrophil mtDNA in SLE pathogenesis.

Apart from self-DNA and ox-mtDNA, the DNA-associated neutrophil microbial peptides LL37 and human neutrophil proteins (HNPs), human beta-defensin 2 and 3 are strong potentiators of IFN responses. LL-37 cause aggregation of DNA fragments, making them resistant to nucleases and facilitating their endocytosis in pDCs via autoantibody-Fc receptor-mediated uptake and IFN production (75, 77, 96). In monocytes, LL37 promoted the uptake of self-DNA to activate type I IFN responses through cytosolic DNA sensor cGAS-STING (79). Overall, in different cell types LL37-DNA complexes are potent inducers of type-I IFN through cytosolic or endosomal sensing. Not surprisingly, 40–55% of SLE patients were also found to develop anti-LL37 and anti-HNP antibodies, which significantly correlated with serum IFNα and disease activity score (77). These data suggest that increased NETosis drives chronic IFN production from pDCs in SLE patients, via production of high molecular weight immune complexes containing gDNA, ox-mtDNA and LL37. It was recently shown in human SLE patients that LL37-DNA complexes from netting neutrophils promoted internalization of self-DNA resulting in activation of LL37-specific human memory B Cells via TLR9 stimulation and production of anti-LL37 Abs (80).

In summary, autoimmune responses to NETs studied so far provide evidence for NET-DNA (gDNA/ mtDNA) as a TLR9 ligand and as an adjuvant promoting IFN production and polyclonal proliferation of B cells, including DNA reactive B cells in SLE, RA (81), psoriasis etc. However, there is little evidence to suggest that NET-DNA serves as a direct autoantigen for DNA-reactive B cells. Further experiments need to be undertaken to answer these questions.

### Intracellular and Apoptotic DNA

Oxidized mtDNA generated within the cells due to oxidative stress can be immunogenic if not processed and purged efficiently. Autophagic clearance of cytoplasmic substrates in the lysosomes has been suggested to prevent the availability of altered self-antigens including modified nuclear-DNA and ox-mtDNA in the cytosol (97, 98). A recent study using monocytes from SLE patients found that autophagic degradation of mtDNA in lysosomes is essential to prevent its accumulation in the cytosol. When accumulated, mtDNA activated the cGAS-STING pathway causing differentiation of monocytes into autoinflammatory DCs (99). Interestingly, IFNα signaling triggered increased mitochondrial respiration, oxidative stress and impaired lysosomal degradation in monocytes, suggesting a direct role of IFNα in autoinflammation (99). This study highlights the importance of efficient mitochondrial recycling through autophagy in the maintenance of peripheral tolerance. In addition to mtDNA, apoptotic DNA internalized by phagocytes is also digested within acidified lysosomes. Inefficient lysosomal maturation in macrophages derived from lupus-prone MRL/*lpr* mice caused increased oxidative stress and impaired acidification of lysosomes. This promoted prolonged accumulation of internalized nucleic acids in endolysosomes and leakage into the cytosol, activating TLRs, and cytosolic sensors (100). Overall, autophagic and lysosomal degradation of self/internalized nucleic acids and associated proteins prevents autoinflammation.

### Microparticles

Apoptotic cells are quickly efferocytosed by macrophages under an anti-inflammatory program, the impairment of which can contribute to SLE (101). Upon cell death, DNA could be exposed extracellularly on apoptotic bodies (102), microparticles (MPs) (70), or as nucleosomes (103). MPs are small lipid membrane bound vesicles of 0.2–1 μm in diameter, generated during late apoptosis/ early necrosis of platelets, leukocytes, endothelial cells, or upon cellular activation through TLRs (70). MPs are decorated with different proteins like transporters, adhesion molecules, surface receptors etc., depending on their cellular parent, along with several constitutive proteins like galectin 3 binding protein (G3BP) (71), HMGB1 (104) and histones. MPs also contain nucleic acids like DNA, RNA, and microRNAs which could be surface exposed or encapsulated (105). MP-associated DNA appears concealed from the most abundant extracellular nuclease—DNase1, and specifically requires the activity of DNase1L3 for efficient degradation (67). Due to their ubiquitous production by all cells and unique structural/ antigenic properties, MPs present the most abundant and enduring source of autoantigens including cfDNA.

Although MPs are produced in all individuals and were proposed to have homeostatic functions (106, 107), several pathologies are also associated with them. Considerable increase in numbers, alterations in cellular origin and composition of circulating MPs have been implicated in atherosclerosis, thrombosis, vasculitis, systemic sclerosis, diabetes, thrombocytopenia, and rheumatoid arthritis (72, 107–109). MP-associated DNA and proteins have also emerged as important contributors to SLE pathogenesis. Antibodies from SLE patient sera and mouse models, as well as monoclonal anti-dsDNA Abs, have been shown to bind DNA in microparticles (67, 73, 110). There is also a significant increase in proportions of MPs in SLE patients with surface bound IgG2, IgM, and C1q, which positively correlates with disease activity, anti-DNA Abs titers and complement activation in patients (110, 111). There is also an increase in the concentration/ proportion of circulating MPs in SLE sera with altered protein composition—expressing VCAM-1, CD40L, HMGB1, or G3BP (71, 110, 112), which could serve to further engage ICs. In agreement, MPs-expressing G3BP were found to predominate in SLE patient sera (*n* = 44) (71). Moreover, colocalization of G3BP with IgG was imaged by immune electron microscopy in the glomeruli of nephritic kidneys, suggesting local cell-derived MPs as additional source of autoantigen for tissue IC-deposition (71). Overall, it is likely that circulating ICs form early in lupus development and initially may not reach the threshold of pathogenicity. Their eventual deposition in tissues and the ensuing organ damage could be enhanced by additional local factors such as impaired degradation of DNA. This multistep process may also explain why not all lupus patients develop lupus nephritis.

It was reported that MP-associated ICs from SLE patients promote ROS production in neutrophils and prime them for LPS-mediated NETosis (113, 114). MPs derived from SLE patients activated blood-derived pDCs and monocyte-derived DCs to express increased CD80, CD83, IL-6, and TNFα (113). Notably, unlike SLE-MPs, MPs from controls, RA and systemic sclerosis patients lacked MP-associated chromatin and did not activate DCs, nor induced NETosis (113). This agrees with our observations that about 1/3^rd^ of the SLE patients with sporadic SLE, have DNase-sensitive chromatin on the surface of their MPs (67). The loss of DNase1L3 activity causes preferential accumulation of DNA in MPs (67) as well as the presence of higher molecular weight DNA in the plasma (90). These higher order structures are much more capable of engaging multiple BCRs in a stable interaction (4), and therefore could be potent stimulators of B cells with DNA-reactive BCRs. Together these studies are suggestive of a significant role of MP-associated chromatin as an abundant source of self-DNA in SLE, for activation of pDCs and DCs via the Fc receptors and potentially direct activation of DNA-reactive BCRs.

### Microbial (Bacterial/ Viral) DNA

SLE is a multifactorial disease requiring genetic susceptibility and environmental triggers for complete loss of tolerance and pathogenic manifestations. A major cause of lupus flares and increased disease activity in SLE patients is due to infections (7). Bacterial infections are most common in SLE patients and thought to contribute to SLE pathogenesis by enhancing inflammation and generating cross-reactive B cells which recognize bacterial as well as self-DNA (61). Bacterial amyloid protein-DNA composites were shown to stimulate a potent IFN response and trigger autoantibody production including anti-dsDNA Abs in lupus-prone as well as wild type mice (115, 116). Infections by all bacterial strains expressing amyloid-DNA complexes could potentially trigger autoimmunity in predisposed individuals, which could contribute to sporadic SLE and also lupus flares.

The role of microbiota in autoimmunity is well-appreciated, although poorly understood (117). A recent study showed that the pathobiont *Enterococcus gallinarum* was able to translocate to the liver and activate autoantigenic T cells, induce IFN-responses through TLR7 stimulation and anti-dsDNA Ab production in lupus prone mice. Accordingly, the pathological responses could be alleviated by antibiotic treatment (85). In several SLE patients, reactivation of human polyomavirus (BK virus) generates antibodies to T-antigen, DNA and DNA-binding proteins—TBP (TATA-box binding protein) and CREB (cAMP response element binding protein). Specifically, anti-dsDNA Ab were confined to patients with frequent polyomavirus reactivations and expression of T antigens (86), indicating a role for T-Ag-DNA complexes in the stimulation of DNA-reactive B cells. Other potential sources of cfDNA in autoimmunity include tumor-derived DNA in cancer patients and fetal-DNA in pregnant females (66, 91). Altogether, these studies suggest that microbial DNA may promote autoimmune responses including the production of anti-DNA Abs; however, its primary antigenic role in the loss of tolerance to self-DNA has not been firmly established.

### DNases as Key Regulators of Immunogenic DNA

Innate nucleic acid (NA) sensors do not discriminate between foreign and self-NAs, hence the processing or metabolism of endogenous NAs is of paramount importance to prevent immune stimulation. Therefore, it is not surprising that ~40% of the genes involved in monogenic or Mendelian-inherited forms of autoimmunity are nucleases. Nucleases can be broadly classified into two main categories depending on their spatial expression: (1) Intracellular nucleases—cleave NAs inside the cells, during apoptosis or after uptake of apoptotic bodies. (2) Extracellular nucleases—cleave NAs exposed extracellularly during apoptosis or generated outside of the cells. The tissue expression profile, structure, enzymatic activity, and functions of the two main classes of DNases in various pathological conditions were recently reviewed (118, 119).

### Intracellular Nucleases: Major Negative Regulators of Autoinflammation

#### Cytosolic Nucleases

Genetic autosomal recessive mutations in RNA processing enzymes of the RNASEH2 complex, *ADAR1*, and *SAMHD1* cause abnormal induction of type-I IFNs and lead to Aicardi-Goutières syndrome (AGS) and related interferonopathies. In addition to these RNases, an autosomal recessive mutation in the cytoplasmic–ER membrane-resident 3′-DNA repair exonuclease1 (TREX1 or DNASEIII) also causes AGS and SLE (120, 121). Classical AGS is identified very early in age, mainly as a neuroinflammatory disorder of the central nervous system with very high levels of IFNα in the cerebrospinal fluid. Glaucoma, thrombocytopenia, hepatomegaly, chilblain-like skin lesions, and late onset of SLE like symptoms are also typical of AGS (122). Analysis of serum autoantibodies from 56 AGS patients (23.4%-*TREX1*; 57.1%-*RNASEH2B*; 2.1% *RNASEH2A;* 4.3%; 8.5% *RNASEH2C;* 4.3% *SAMHD1*; and 4.3% *ADAR1* mutants) was performed, using an autoantibody array to assess their antigen-specificity. The study revealed their specificity to nuclear antigens like gp210, PCNA, Ro/SSA, Sm/RNP, SS-A/SS-B etc. Even though AGS and SLE share several overlapping disease manifestations, ss/dsDNA specific antibodies were not detected in any of the AGS patient sera in this study (123). Moreover, in a previous AGS clinical study, only 3 patients (all <3 years age) from a cohort of 24 had anti-dsDNA Abs. Among the three, one patient had a mutation in *TREX1*, one in *RNASEH2C* and one had an unknown mutation (124). *Trex1*^−/−^ mice do not develop classical AGS, but rather develop lethal inflammatory myocarditis, without anti-chromatin/ DNA Abs (125, 126). These studies indicate a limited role for the intracellular exonuclease *TREX1* in anti-DNA B cell responses.

### Lysosomal DNases

DNASE2 is an endonuclease that functions in the lysosomes and is known to process DNA internalized with apoptotic cells. DNASE2 is expressed by macrophages in almost all tissues. Mice deficient in *DNASE2* die *in-utero*, due to an overwhelming IFNα response and lethal anemia (127, 128). Sequencing analysis on 24 SLE patients from a Korean cohort revealed 6 sequence variants of *DNASE2*, all of which were at a higher risk for renal disorders but showed no significant association with SLE (129). Recently, three individuals from two families of Algerian or Italian ancestry were identified with biallelic mutations in *DNASE2*, causing complete loss of DNASE2 endonuclease activity. They were able to survive with medical intervention but had severe neonatal anemia, glomerulonephritis, liver fibrosis, and arthropathy. The hallmark yet again was the excessive production of IFNα and associated interferonopathies (130). Remarkably, all the patients with *DNASE2* mutations had high titers of anti-DNA Abs and renal disorders. Further analysis of DNASE2 in SLE patients will shed more light on its role in SLE pathogenesis and anti-DNA responses. Notably, both the intracellular DNases—TREX1 and DNASE2, signal through the cGAS-STING pathway for IFN production (131).

Most recently, two endolysosomal proteins phospholipases D3 and D4 (PLD3/PLD4) with putative phospholipase activity were shown to have a functional 5′ exonuclease activity preferentially on unstructured ssDNA. PLD3 or PLD4-deficient mice displayed a TLR9-stimulated inflammatory syndrome while PLD3/4 double-deficient mice were unable to survive beyond the age of 21 days due to severe liver inflammation. Interestingly, the observed autoinflammatory syndrome was mediated by IFNγ instead of IFNα. Although there was excessive TLR9 activity causing IFNγ production, no autoantibody responses were reported (132). Polymorphisms in PDL4 linked to RA and systemic sclerosis (133, 134) have also been reported. Altogether, these studies identify the predominant function of intracellular nucleases in preventing autoinflammatory conditions, whereas their contribution toward anti-DNA antibody responses may be limited, as shown in Figure 1.

### Extracellular DNases: Negative Regulators of Extracellular Immunogenic DNA

#### DNase1: A Potential Negative Regulator of Lupus Nephritis?

DNase1 is the most abundant secreted endonuclease, that is primarily expressed in the salivary glands, kidneys and gut (135). The association of DNase1 with SLE was initially identified through the *DNase1*^−/−^ mouse model generated on a mixed B6/129 background, in which some mice developed anti-DNA and anti-nucleosome-Abs (predominant), as well as glomerulonephritis in a gender-independent manner. However, in subsequent studies it was shown that the B6/129 mixed background itself caused most of the observed SLE phenotype (136), as *DNase1*^−/−^ mice on a pure B6 background did not develop SLE features (68).

Similarly, limited association of DNASE1 with anti-DNA antibody production in human SLE has been identified. Two Japanese patients that developed serological features of SLE with high titers of anti-DNA and anti-Nuc Abs, were identified with an A → G mutation in exon 2 of human *DNASE1* causing a 3–4 fold reduction in enzymatic activity (137). Till date there are no further reports on SLE patients identified with similar or other mutations in *DNASE1* (138). However, in another study with 113 SLE patients, Dnase1 activity was found to be significantly lower in SLE patients compared to healthy controls, which negatively correlated with anti-Nucleosome antibody titers. No correlation was found between reduced DNase1 activity and SLE disease flare-ups or kidney complications in this cohort (139). Notably, kidney biopsies from 10 patients were screened for DNASE1 activity of which 4 patients had SLE-associated nephropathy. These 4 patients showed a concurrent low enzymatic activity of DNASE1 compared to healthy controls (140). In agreement with these observations, a reduction in DNASE1 expression in kidney, and urine directly correlated with progression of lupus nephritis in mouse and in humans with self or transplanted kidneys (141, 142). These studies suggest a potential role of locally produced DNase1 in the prevention of immune complex deposition and subsequent kidney nephritis (143).

Unlike its role in anti-DNA responses, the function of Dnase1 in the degradation of NETs is better established. Healthy human serum was able to degrade NETs *in vitro* and the functional component was identified to be DNase1. Conversely, sera from 36.1% of 61 SLE patients had poor or no ability to degrade NETs *in vitro*. These patients were found to have high anti-NET Abs which hampered the accessibility of DNASE1 to NETs, Notably, in this cohort of SLE patients the poor NET degraders had severe kidney nephritis (94)—further supporting a potential role of DNase1 in preventing kidney nephritis. Recently, NET degradation activity of Dnase1 was further corroborated when Dnase1 was also shown to have a redundant function along with DNase1L3 in the degradation of NETs formed during sterile neutrophilia and septicemia. The absence of DNase1 and DNase1L3 both caused vascular obstruction and organ damage. The results were consistent with human sera samples and in two different mouse models of DNase1 deficiency (82). This chromatin-degrading effect of Dnase1 seems to be specific to NET-associated DNA as the quality and quantity of cfDNA fragmentation was indistinguishable between plasma from WT and *DNase1*^−/−^ mice (144). Overall, the available data from SLE patients and *DNase1*^−/−^ mice do not indicate its involvement in anti-DNA antibody responses, whereas its role in SLE-related renal pathogenesis is prominent and deserves further exploration.

#### DNase1 Like 3: Major Negative Regulator of Anti-DNA Responses

As the name indicates, DNase1L3 bears close structural and functional resemblance with DNase1 and together they comprise the secreted endonucleases in the serum (145). DNase1L3 is one of the family members of three homologous DNase1 like proteins. The DNase domain of all the Dnase1 family of enzymes is highly conserved, while the C terminal domains are most variable and may impart unique attributes to the enzymes. DNase1 and DNase1L2 lack a C-terminal domain, while DNase1L1 has a GPI-anchored hydrophobic region. DNase1L3 contains a positively charged C terminal domain (146). Homology modeling suggested that the C-terminal peptide of DNase1L3 may stretch out at a fixed angle from the main DNase domain with a stable α-helical secondary structure bearing a positive charge (67). Upon deletion of the C-terminal domain or modulation of its conformation, DNase1L3 lost its unique abilities of (1) efficiently degrading DNA within polynucleosomes and (2) digesting liposome-coated DNA (67, 147). Although the exact mechanism by which the C-terminal domain of DNase1L3 imparts the protein its unique ability to access lipid-encapsulated and histone-protected DNA is not clear, the positive charge on the α-helix may facilitate lipid membrane binding/ penetration and dislocation of histones from DNA. This unique structural property of DNase1L3 poises it to digest MP-associated DNA and prevent accumulation of extracellular DNA, thereby suppressing SLE. Indeed, we found that IgG from sera of at least 2/3rd of the 53 patients with sporadic SLE, bound to the surface of MPs. Pre-treatment of MPs with DNase1L3 abolished this binding in half of the patients indicating that the IgG binding on the surface of MPs was DNase1L3-sensitive (67). This finding could have implications in using DNase1L3 as a therapeutic to reduce MP-DNA-dependent immune complex formation. DNase1L3 was also recently shown to degrade intravascular NET-DNA and prevent vascular occlusion by disrupting NET clots similar to DNase1 (82).

The role of DNase1L3 in autoimmunity was discovered during clinical human patient analysis, summarized in Table 2. A homozygous 1-bp deletion in *DNASE1L3* (c.643delT) caused pediatric-onset familial SLE (148). Homozygous frameshift mutations—c.289_290delAC and c.320+4delAGTA in *DNASE1L3* led to exon skipping and pediatric SLE in two respective families (149). Recently, four Italian affected individuals were identified with similar mutations in *DNASE1L3* (150). In addition to null mutations, SNPs have also been reported in DNASE1L3 in humans. A heterozygous SNP C686/T686 resulting in R206C substitution in DNASE1L3 was found to reduce the DNase1L3 enzymatic activity (151). Furthermore, SNPs in DNase1L3 gene have also been associated with a related autoimmune disease Scleroderma (152, 153). Similar to humans, DNase1L3-deficient (DNase1L3KO) mice (on a pure B6 or pure129 background) develop SLE-like symptoms, including the gender-neutral formation of high titers of anti-DNA abs at an early age (67). Recently, another strain of DNase1L3KO mice was shown to develop anti-DNA responses that were further enhanced in SLE-prone mice (154). In striking contrast with all other nucleases, the anti-DNA responses in DNase1L3KO mice were STING-independent but MyD88-dependent (67). These data identify DNase1L3 as a DNase that is uniquely structured to access and degrade DNA associated with lipids and DNA-binding proteins. It forms an essential component of the DNase arsenal, in the absence of which extracellular DNA escapes degradation and can be a direct autoantigen for the activation and proliferation of DNA-reactive B cells. In agreement, DNase1L3-deficient patients and DNase1L3KO mice show the development of anti-DNA Abs at a very early age.

**Table 2:** Known cases of *DNASE1L3* mutations in human subjects.

	**Cohort details**	**Identified mutations in *DNASE1L3***	**Disease characteristics**	**Reference**
1.	6 families 17 affected subjects (6 Females. 11 males)	Homozygous 1-bp deletion c.643deiT	ANA^+ve^, Anti-dsDNA^+ve^, ANCA^+ve^ Hypocomplementemia Nephritis in 10 subjects SLE in all subjects with SLEDAI: 8-22	(148)
2.	2 families 5 affected subjects (All females)	Homozygousframeshift mutation. c.289_290deiAC and c.320+4deiAGTA	HUVS in all subjects SLE in 4 subjects ANA^+ve^, Anti-dsDNA^+ve^, ANCA^+ve^ Hypocomplementemia Nephntis(classll-111) in 3 subjects	(149)
3.	1 family 4 affected subjects (2 females. 2 males)	Homozygous 2b frameshift deletion c.289_290deiAC	ANA^+ve^, Anti-dsDNA^+ve^, ANCA^+ve^ Polyarthritis Glomerulonephritis Vasculitis Hypocomplementemia	(150)
4.	9 populalons >90 subjects per group	Heterozygous SNP C686fT686 resulting in R206C substitution Found mainly in European Populations	Reduced Dnase1L3 enzymatic activity Disease association not studied	(151)

Altogether, as depicted in Figure 1, these studies on nucleases provide evidence that (1) Anti-DNA responses are not induced by excessive IFN production *per se*, arguing that IFNs play a major role in the amplification of anti-DNA responses but not in the breakdown of tolerance to DNA. (2) Extracellular availability of immunogenic DNA as a direct antigen drives anti-DNA reactive B cell responses in SLE, making a strong case for the regulatory role of extracellular DNases in anti-DNA antibody production.

### Nucleic Acid Sensors: The Double-Edged Sword

It is now well-established that microbial NA sensors also recognize self-NAs under autoimmune conditions (69, 155, 156). For that reason, self-NA availability to NA-sensors is limited by nucleases and the availability of several NA-sensors is stringently controlled by their localization inside endosomes and by post translational processing for function (157). Together, they prevent self-NA availability and sensing. The contribution of DNA and RNA-sensors in autoimmunity has been the topic of several comprehensive reviews (158, 159). Here we highlight some key points related to the DNA-sensors involved specifically in the antibody response to DNA. We discuss literature which emphasizes the prominent role of endosomal TLRs in DNA sensing to generate an “autoantibody response,” unlike the cytosolic DNA sensors which mainly engage an “autoinflammatory response.” All the DNA and RNA-sensors are intracellular and further divided into two groups—cytosolic or endosomal. In keeping with the main theme of this review, we discuss the known NA-sensing pathways regulated through intracellular and extracellular nucleases.

### NA-Sensing Regulated Through Intracellular Nucleases

Mice deficient in RNaseH2 complex proteins, SAMHD1 and ADAR1 are either embryonically lethal or do not recapitulate human AGS. Yet, they revealed that RNaseH2 or SAMHD1-dependent NA-accumulation led to the stimulation of the cGAS-STING pathway, while loss of ADAR1 (deaminase) stimulated the RNA sensing MDA5-MAVS pathway (118). Cytosolic DNA sensing due to DNaseIII or Trex1 deficiency stimulated the cGAS → cGAMP → STING → IRF3 → IFNα signaling axis (121, 126, 131). Overall, the predominant response to DNA sensing in the cytosol was autoinflammatory in both humans and mice. Additionally, although antibodies with other specificities were observed, DNA specific antibodies were detected only in a minority of patients (118, 122, 124). As highlighted in Figure 1 in green, these studies point toward the role of cytosolic cGAS-cGAMP-STING signaling pathway in autoinflammation but not in the initiation of anti-DNA antibody responses.

The contribution of lysosomal DNase2 in anti-DNA antibody production is complicated partly because of the absence of a viable mouse model and partly due to the paucity of human patients identified with *DNASE2* mutations. The absence of DNase2 in mice causes accumulation of apoptotic cell derived DNA in the lysosomes of liver and bone marrow macrophages causing lethal anemia and cell death. DNase2KO mice can be rescued by the deletion of IFNAR. However, “IFNAR-deficient DNase2KO mice develop chronic polyarthritis, splenomegaly, and ANA. The accumulated DNA stimulates the cGAS-STING-IRF3/7 pathway leading to massive type-I interferon production, because the deficiency of either cGAS (131)/ STING (160, 161)/ IRF3 or IRF7 (162)/AIM2 (161) can rescue the mice form prenatal anemia and severe arthritis. While the generation of IFNα and TNFα in DNase2-deficient mice as well as humans is documented, anti-DNA antibody production in DNase2KO mice is not predominant (131, 160, 163). Instead, DNase2-IFNAR-double-deficient mice developed antibodies against RNA-associated antigens and splenomegaly in an RNA-driven TLR-dependent manner (161, 163, 164). Further analysis of DNase2 in anti-DNA responses is required as the three DNASE2-deficient patients (age 11–17) reported thus far had fluctuating significant elevations in circulating anti-DNA Abs (130). It is possible that the formation of anti-DNA Abs in these patients and mice is an “after-effect,” as a result of polyclonal activation of B cells due to chronic inflammation and IC formation.

Altogether, the current literature suggests that the cytosolic DNA is detected primarily through the cGAS-STING pathway which induces a potent autoinflammatory response, while the minimal anti-DNA antibody production seems to be a secondary effect. This agrees with the seemingly confounding role of STING in autoimmunity. STING appears to be a potentiator of autoimmune responses by inducing IFNα and downstream ISGs (165, 166). Remarkably, patients with overactive STING do not develop detectable anti-DNA Abs (165, 166)—strengthening the idea that cytosolic STING signaling pathway is not directly involved in anti-DNA responses. Contrary to its autoimmune-stimulatory activity, STING-deficiency was shown to exacerbate autoimmune manifestations including anti-DNA antibody production in MRL. *Fa*s^*lpr*^ lupus mouse model (167). It is likely that when cytosolic DNA is absent STING functions as a negative regulator of endosomal TLR signaling through yet undiscovered mechanisms. Alternately, when cytosolic DNA is present STING induces IFNα signaling and autoinflammation. Overall, what we can conclude with confidence is that the cGAS-STING pathway does not seem to induce anti-DNA Ab production.

### NA-Sensing Regulated Through Extracellular Nucleases

As discussed earlier, DNase1 deficiency does not seem to induce anti-DNA responses by itself. However, its local functions reported in the kidney may promote formation of immune complexes laden with DNA, which are known to engage the endosomal TLR9-MyD88 signaling pathway (168). Further studies on DNase1 need to be performed to establish its NA-sensing partners.

DNASE1L3-deficient patients and DNase1L3KO mice both develop high titers of anti-DNA Abs at an early age, without a female gender bias. Using DNase1L3KO mice deficient in either STING or MyD88 we found that anti-DNA and anti-nucleosome responses in DNase1L3KO mice are dependent on the TLR-MyD88 pathway. Further studies are underway to delineate which MyD88-dependent TLRs are involved in the autoimmune responses to MP-associated DNA in D1L3KO mice. Some studies suggest that DNase1L3 may have intracellular localization with functions in apoptosis and inflammation, reviewed in Keyel PA (119). However, as autoimmunity in DNase1L3KO mice is independent of the cytosolic DNA sensor STING (67), it indicates that the anti-DNA responses are specifically due to the extracellular DNase function of DNase1L3. Indeed, we were able to temporarily reduce the anti-DNA Ab titers in DNase1L3KO mice by transient replenishment of circulating DNase1L3 enzyme (67). Overall, extracellular DNases predominantly regulate the stimulation of TLR-MyD88 pathway of DNA sensing, highlighted in yellow in Figure 1.

### Role of Toll-Like Receptors in Anti-DNA Antibody Production

Deficiency of the adaptor protein MyD88 ameliorates SLE specific autoantibodies and associated pathology in several lupus mouse strains (169). Expression levels of TLR2, TLR7, TLR9, IFN-alpha, and LY6E (Sca-1) mRNAs in SLE patients are significantly higher than healthy controls, indicating contribution of TLR-MyD88 signaling pathways in the pathogenesis of human lupus (170). Several studies show that circulating DNA-containing ICs correlate positively with anti-dsDNA Ab production in SLE patients (171, 172). Indeed, the importance of BCR/TLR–dual signaling in autoimmune B cell responses was originally identified by using IC-mediated activation of IgG2a-reactive murine AM14 B cells (69). However, it is now clear that TLR engagement also promotes activation, proliferation/ differentiation of B cells that directly bind DNA (or other autoantigens) through the BCR and may therefore play an important role in the early stages of autoantibody production. Among the MyD88-dependent TLRs—TLR7 and TLR9 are the prominent ones involved in the development of anti-DNA Abs in mouse models of lupus and altered TLR7 and 9 expression has been reported in human SLE patients as well (173). Perhaps the most convincing evidence for the involvement of TLR7/9 signaling in B cells for anti-DNA antibody production comes from the case studies of SLE patients that develop an antibody deficiency syndrome similar to common variable immunodeficiency (CVID). These patients have a complete remission from SLE, with absence of anti-DNA Abs, and B cells are unresponsive to TLR7/9 stimulation—indicating the crucial role of TLR7/9 mediated B cell responses in SLE (174, 175).

### TLR7- the Master of RNA-Driven SLE Pathogenesis

The seminal role of TLR7 in SLE pathogenesis is firmly established. TLR7 promotes the formation of autoantibodies against RNA and RNA-associated proteins. Deletion of TLR7 reduces anti-SmRNP and other RNA-associated antibody responses, however, in most cases there is no reduction in anti-DNA responses (158, 176, 177). It was shown that B cell-intrinsic TLR7 signaling is essential for the formation of spontaneous germinal centers (178). Therefore, in the B6. *Sle1b* lupus mouse model where autoantibody formation is driven predominantly through the GC-pathway (179), TLR7 deficiency also reduces anti-DNA Abs (178, 180). Most-importantly, deficiency of TLR7 ameliorates SLE-associated splenomegaly and nephritis (176, 181), while expression of an extra copy of TLR7 exacerbates it (182). Altogether, TLR7 is the master regulator of RNA-driven TLR-dependent systemic autoimmune manifestations. However, it does not seem to play a direct role in anti-DNA antibody production and yet appears indirectly involved in SLE though its functions in molecular pathways necessary for antibody production and inflammation.

### The Dichotomous Pathogenic and Tolerogenic Role of TLR9 in Autoimmunity

By far the most confounding endosomal TLR is TLR9, which is endowed with both pathogenic and regulatory functions. It is required for the generation of high-titer antibodies to DNA and DNA-associated proteins in several murine lupus models including MRL/*Fa*s^*lpr*^, B6. *Sle1b* and FcγRIIB^−/−^ (158, 169, 176, 178). However, even though anti-DNA specific B cells and antibody titers are reduced in the absence of TLR9, there is an exacerbation of lupus pathogenesis (splenomegaly, nephritis, etc.) and an increase in autoantibodies against RNA-associated antigens (176, 177, 181, 183, 184)—suggesting a negative regulatory or tolerogenic role for TLR9 in the pathogenesis of lupus, by suppressing TLR7-mediated autoimmunity. Similar regulatory role of TLR9 was demonstrated in pristane-induced murine lupus. Pristane exposed TLR9-deficient BALB/c mice had an exacerbated production of autoantibodies against RNA, neutrophil cytoplasmic antigens, and myeloperoxidase and worse renal disease than TLR9-sufficient mice (185). TLR9 has also been shown to promote production of protective IgM antibodies by B-1b cells and prevent expansion of proinflammatory Th17 T cells, thereby regulating systemic autoimmunity (186). Recently, another potential mechanism contributing to the regulatory role of TLR9 was described. Exposure of phagocytes to apoptotic cell-associated DNA (a common antigenic source in experimental lupus), upregulated the expression of the transcription factor AhR (aryl hydrocarbon receptor) in a TLR9-dependent manner. AhR in turn drove the production of the immunoregulatory cytokine IL-10. Therefore, loss of TLR9 or AhR in lupus prone mice exacerbated disease (187). These studies indicate that TLR9 stimulation by DNA in macrophages could be immunosuppressive. However, B cells may not be subject to the same suppressive effects as they are poor phagocytes and this aspect needs further investigation.

### A Potential Role of Surface TLRs in Anti-DNA Responses

Several studies indicate an indirect contribution of cell surface TLRs in anti-DNA antibody production through HMGB1 which is a DNA binding protein known to stimulate TLR-signaling and induce a proinflammatory program (188). In C57BL/6^lpr/lpr^ mice deficient in TLR2 or TLR4, glomerular IgG deposition and mesangial cell proliferation were remarkably decreased, and ANA, anti-dsDNA, and anti-cardiolipin autoantibody titers were significantly reduced (189). Moreover, TLR2-deficiency significantly reduced anti-DNA/nucleosome antibodies, renal disease, and IL-6 production in a pristane-induced lupus mouse model (190). Similarly, in SLE patients, HMGB1 in circulating DNA-containing ICs from SLE patients induced production of anti-dsDNA Abs through the TLR2-MyD88 pathway *in-vitro* (172). Recently, amyloid curli-DNA complexes were also shown to stimulate TLR9 via TLR2 (87).

In summary, the studies so far suggest a central role for TLR9 in the induction of anti-DNA antibody responses, supported by TLR7, 2, and 4. TLR9 is the only direct endosomal DNA sensor and induces a robust IFN response upon stimulation. However, its endosomal localization limits its accessibility. Therefore, IC-mediated internalization through Fc-receptors or direct BCR mediated uptake of DNA are the most potent inducers of TLR9 and anti-DNA Abs. Surface TLRs might play a role in aiding the delivery of DNA to TLR9. However, for long-lived plasma cell formation the germinal center pathway of differentiation is necessary. TLR7 plays a key role in the formation of spontaneous GCs, probably via stimulation through endogenous retroelements (191). Therefore, TLR7 signaling could further promote anti-DNA responses via the GC-pathway. It is more likely that for an effective anti-DNA B cell response all these TLRs—TLR9, TLR7, and TLR2/4 are required in tandem and the inflammatory program induced by them which includes cytokines like IFNα, IFNγ, IL-6, IL-10, and TNFα is necessary for the amplification of DNA-reactive B cells.

### Interferons: Key Effectors in the Development and Progression of Anti-DNA Responses and SLE Pathogenesis

#### Type-I Interferons: Prominent Role of IFNα in Anti-DNA Responses

The three main pathways of type-I interferon induction include sensing by (1) cGAS-STING, (2) RIG-I/MDA5-MAVS, and (3) TLR-MyD88/TRIF. Their involvement in the pathogenesis of several rheumatic diseases and the current therapeutic interventions targeting the type-I interferon pathway have been extensively discussed in excellent reviews elsewhere (192, 193). Type1 interferons are at the core of several disease manifestations in monogenic disorders discussed above, so much so that most of the symptoms have been classified separately as “type-I interferonopathies,” with AGS as the prototype. Loss of function mutations in cytosolic DNA/RNA processing enzymes and gain of function mutations in the ds-RNA receptor gene IFIH1 (MDA5) (194, 195) and adapter protein *TMEM173* (STING) cause excessive type-I IFN production leading to severe disease pathologies (122, 196). However, as discussed in previous sections, the induction of type-I interferons by the stimulation of cytosolic NA sensors does not seem to engage the pathways directly responsible for anti-DNA antibody production, although it is crucial for disease manifestations with features overlapping that of rheumatic diseases.

On the other hand, accumulating evidence implicates the TLR pathway of type-I IFN production as a major pathway of anti-DNA Ab production. The most convincing data is from SLE patients with genetic variations in the major proteins involved in the TLR-IFN signaling axis. Polymorphisms in TLR signaling pathway proteins such as IRF5, IRF7, IRF8, and IRAK1 are associated with SLE (197, 198). Most of the polymorphisms in IRFs directly correlate with high anti-DNA and anti-Ro/La/Sm antibodies which result in increased IFNα activity (199–201). These studies suggest a crucial role of autoantibody-DNA/ RNA complexes in the stimulation of the TLR pathway resulting in increased type I IFN production (Figure 1), which feeds into an amplification loop. Although the role of ICs in the production of type-I IFNs is clear, whether they are directly involved in the generation of autoantibodies found in those ICs is less understood. In this regard, understanding the direct vs. indirect effects of type-I IFNs on B cells may be critical. Indeed, a number of studies have looked at the effect of IFNα on B cells in various lupus-prone mouse models by overexpressing IFNα through adenovirus in NZB/W-F1, NZW/BXSB and B6. *Sle1,2,3*, reviewed in Liu et al. (202); or by deleting the IFNα receptor in B cells of B6. *Sle1b* (203) and WASp-chimeric (204) experimental lupus models. In all these models IFNα signaling positively correlated with B cell activation and differentiation into GC B cells and antibody-forming cells and high anti-DNA Ab titers. In the B6. *Sle1b* model, IFNα specifically promoted autoreactive B cell expansion and positive selection through the germinal center pathway but was dispensable for B cell responses against foreign antigens (203). Interestingly, in most experimental models, IFNα production through the TLR7 signaling pathway seems to play a major role in SLE by either regulating germinal center formation or by promoting the generation of IC-forming pathogenic autoantibodies that ultimately cause kidney pathology. TLR9-deficieny in MRL.Fas(lpr) mice caused exacerbated renal disease which was abrogated in the absence of IFNAR-signaling through specific reduction of anti-RNA specific antibodies (205), suggesting a crucial role of TLR7-IFNα signaling axis in SLE pathogenesis. In DNase1L3-deficient mice where increased accumulation of undigested cfDNA leads to specific production of anti-chromatin and anti-DNA antibodies through the TLR-MyD88 pathway, IFNα overexpression exacerbated anti-DNA responses and mortality (67). Collectively, these studies establish an important role of type I IFN in autoantibody-driven inflammation, although it has been difficult to distinguish its effects on autoantibody production *per se* vs. the downstream inflammatory process.

#### IFN Gamma: Initiator of Anti-DNA Antibodies in SLE?

Type-II IFN (IFNγ) has been implicated in both human and mouse lupus (206). Accumulation of autoantibodies has been shown to precede clinical presentation of SLE disease by several years (2). A comprehensive longitudinal analysis of lupus autoantibodies, IFNα, and IFNγ from serum samples of 55 patients before and after clinical onset of SLE, with matched controls was performed. The study revealed that in SLE patients, autoantibodies appear years before clinical SLE is detectable but notably they either coincided with or followed an increase in IFNγ. In contrast, increase in IFNα was observed mostly at the time of detectable clinical disease (207). These findings suggest IFNγ as the initiator and IFNα as the propagator of autoantibody production. In line with this model, recently, in two independent studies with B6. *Sle1b* or WASp murine models of lupus, deletion of IFNγ receptor in B cells led to a complete loss of germinal centers, abolishment of anti-dsDNA Abs and systemic autoimmune manifestations (204, 208). In WASp chimeric mice and in human B cells, IFNγ signaling along with BCR/CD40 and TLR signaling was shown to be necessary for the induction of Bcl-6, the master regulator of GC responses (204). Alternately, in the B6. *Sle1b* model, IFNγ signaling through STAT1 was required for the expression of transcription factor T-bet, and IFNγ production in B cells, which then differentiated into pathogenic anti-DNA IgG2b/2c producing cells (208). Interestingly, a subset of B cells expressing T-bet, CD11c, and IFNγ named age-associated B cells (ABCs), have been implicated in SLE pathogenesis as well (209). Moreover, upon deletion of IFNγ a dramatic decrease in anti-dsDNA antibodies of IgG2a subclass and reduced proliferation of B cells was also observed in the MRL-FAS (lpr) mice (210), where anti-DNA antibody production occurs mainly through the extrafollicular pathway (20). Overall, these studies highlight the important role of IFNγ signaling in the GC, extrafollicular, and ABC-associated pathways of anti-DNA antibody production. Several studies describing IFNγ in SLE, highlight the role of T cells in the production of pathogenic IFNγ (204). Indeed, a higher percentage of CD4^+^ and CD8^+^ T cells from SLE patients produce excessive IFNγ (211). Additionally, Tfh mediated autoimmunity in *Roqui*n^*san*/*san*^ mice was induced by the accumulation of excessive IFNγ producing T cells due to delayed degradation of IFNγ mRNA (212). Considering the evident importance of IFNγ in autoimmunity, neutralization, or reduction of IFNγ has been tried as a therapeutic modality in mice and human SLE. Significant reduction in autoimmunity was observed in NZB/NZW-F1, MRL. *Fa*s^*lpr*^ and pristane induced mouse model of lupus, however, due to the crucial role of IFNγ in antiviral immunity in humans, the usefulness of IFNγ blockade in SLE may be limited (206).

Overall, the role of interferons in anti-DNA Ab production is well-established. However, due to the significant overlap between the genes induced by IFNα and IFNγ (213, 214), it is harder to delineate their individual contribution to autoantibody production and SLE pathogenesis. Based on the evidence discussed above, it is plausible that under autoimmune conditions (in the presence of cfDNA/RNA or cell-intrinsic defects), autoantibody production is initiated by the stimulation of B cells by antigen-stimulated IFNγ-producing T cells, leading to autoreactive B cell proliferation and differentiation. The autoantibodies produced thereby form complexes with the cfDNA/RNA may then promote pDC/DC activation and IFNα production, which would further amplify the response, as shown in the schematic in Figure 1.

## Conclusions

Emerging genetic and functional evidence suggests that the efficient degradation of extracellular DNA is an important checkpoint in preventing the stimulation of DNA-reactive B cells. As depicted in Figure 1, a B cell-intrinsic TLR-MyD88 pathway of DNA recognition seems to be necessary for the break in tolerance to DNA, supported by helper T cells recognizing DNA-associated antigens. These autoreactive B cells could further proliferate and differentiate in response to type-I IFNs produced by themselves and by myeloid/ stromal cells, thereby amplifying the autoantibody response. Therefore, using interventions that could enhance or amplify the degradation of extracellular DNA may work to impede the production of anti-DNA antibodies and could be tested as a therapeutic for SLE.

## Author Contributions

All authors listed have made a substantial, direct and intellectual contribution to the work, and approved it for publication.

### Conflict of Interest Statement

The authors declare that the research was conducted in the absence of any commercial or financial relationships that could be construed as a potential conflict of interest.
